# Medical Congress—Dental Section

**Published:** 1887-10

**Authors:** J. Taft, W. Mitchell


### Medical Congress.—Dental Section.

The Ninth International Medical Congress is a thing of the past. Its meeting was an occurrence that elicited much interest for more than two years previous. The results attained are perhaps all that could have been expected, and especially when the embarrassments that attended the organization are taken into account. Determined opposition was manifested by many leading men in the medical profession during almost the entire time since the meeting of the Eighth Congress. But notwithstanding all this, the measure of success attained was very gratifying indeed. This is the general expression in regard to all the sections of the congress. For others we can not so definitely speak as for that of Dental and Oral Surgery. This in all its parts was in its realization doubtless equal to, if not beyond, the highest expectations entertained for it.

Quite a difference of opinion was entertained by many in the dental profession for a long period in reference to the propriety of holding such a section. On this account there was, of course, not the full co-operation and effort that would otherwise have been given.

While we accord to every one entire freedom of opinion and views, many have experienced regret that there could not have been full and harmonious action and co-operation from the beginning of the organization of this section. But, notwithstanding, a large measure of success was attained, and this was due not to the efforts of a few individuals, but to the concurrent effort and labor of a large number in the profession. In the work of the section the utmost harmony prevailed throughout. Every one upon whom duty devolved performed his work well and thoroughly, giving special attention to that which he had in hand. In the different practical departments this was eminently true, and as a result, never before were there such clinical and prosthetic demonstrations given.

In the general sessions of the Section, of which there were ten, the utmost harmony and order prevailed. The work was systematically arranged and as perfectly carried out. The greatest interest was manifested in the work done in the section, and probably never before was there greater interest given to the work nor more earnestness in its performance than was realized during those five days. The interest continued unabated to the very hour of closing. There was very little routine or miscellaneous work to interrupt the regular current of business.

It was the delight of all our professional brethren of this country that so many members of the profession from abroad were present and joined so heartily in the work. This was really one of the very gratifying features of the occasion, and we are encouraged to hope that they shared to the full with us of the good things of the occasion. They brought us much that was new, interesting, and valuable; and may we not hope that they received something in return?

And now, as one who felt a great interest in and shared much of the feeling of responsibility for the work done, I can not do less than extend most hearty thanks to all who contributed to the success of the Section; for to the earnest workers who gave so much time, attention, and labor must be attributed in a very great measure the success, and may they each and every one have the satisfaction of feeling and knowing that they have contributed very greatly to the success and profit of this memorable occasion. J. TAFT.

---

The following letter will be of interest to the readers of the REGISTER, inasmuch as it makes quite plain a matter about which there has been some doubt, especially in regard to the status of American diplomas in England. Quite a step has been recently taken in England for the advancement of dental education by the application of a section of the dental law that has hitherto been inoperative, by virtue of the enforcement of which English dentists, after June 1st, will be required to pass the conjoint examinations of the colleges of physicians and surgeons, as well as the examination for the L.D.S. But the extract from the letter will speak for itself, and is as follows:

EDITOR OF REGISTER:—*Dear Sir:*—You must pardon my seeming negligence in failing to reply to your letter sent by my brother. The reason of the delay was that I desired to furnish you with correct data respecting the effect of the lately applied law that came into effect June 1st. That only applies to English dentists. That is, after June 1st, all English dentists will be required to pass the conjoint examinations of the colleges of physicians and surgeons as well as the examination for the L.D.S. And those who though having been in practice ten years prior to the passage of the Dental Act, and have failed to register prior to June 1st, will be considered as practicing illegally. The position of the University of Michigan and Harvard, as regards the eligibility of their dental diplomas for registration, remain unchanged; and there seems to be no desire on the part of the Medical Council to at all interfere against them; their ideas toward these institutions being the same as when they decided them eligible to registration, feeling satisfied with the exactions of the curriculum of these Colleges.

Yours sincerely, W. MITCHELL."

